# Knee Strength Assessment and Clinical Evaluation Could Predict Return to Running after Anterior Cruciate Ligament Reconstruction Using Patellar Tendon Procedure

**DOI:** 10.3390/ijerph192013396

**Published:** 2022-10-17

**Authors:** Marc Dauty, Pierre Menu, Pauline Daley, Jérôme Grondin, Yonis Quinette, Vincent Crenn, Alban Fouasson-Chailloux

**Affiliations:** 1 Service de Médecine Physique et Réadapatation Locomotrice et Respiratoire, CHU Nantes, Nantes Université, 44093 Nantes, France; 2 Service de Médecine du Sport, CHU Nantes, Nantes Université, 44093 Nantes, France; 3 IRMS, Institut Régional de Médecine du Sport, 44093 Nantes, France; 4 Inserm UMR 1229, Regenerative Medicine and Skeleton, RMeS, Nantes Université, 44042 Nantes, France; 5 Clinique Chirurgicale Orthopédique et Traumatologique, CHU Nantes, Nantes Université, 44000 Nantes, France

**Keywords:** knee, ACL tear, surgery, patellar tendon graft, strength, running, sport

## Abstract

Background and objectives: Muscle knee strength is a major parameter that allows return to running. Isokinetic strength parameters may predict return to running 4 months after ACLR using the bone–patellar–tendon–bone procedure. Materials and methods: The isokinetic knee strength of 216 patients (24.5 ± 5 years) was measured 4 months after surgery, and progressive return to running was allowed. The effectiveness of return to running was reported at 6 months. Return to running prediction was established using multivariate logistic regression. Predictive parameters were presented with a ROC curve area to define the best cut-off, with sensibility (Se) and specificity (Sp). Results: A model was established, including the limb symmetry index (LSI), and 103 patients (47.6%) were able to run between the fourth and the sixth month after surgery. These patients presented significantly fewer knee complications, a better Lysholm score, a better Quadriceps and Hamstring LSI and better quadriceps strength reported for body weight on the operated limb. The best model was established including the Quadriceps and Hamstring LSI at 60°/s and the Lysholm score. The cut-off for Quadriceps LSI was 60% (ROC curve area: 0.847; Se: 77.5%; Sp: 77%), for Hamstring LSI 90% (ROC curve area: 0.716; Se: 65.7%; Sp: 60.2%) and for Lyshom score 97 points (ROC curve area: 0.691; Se: 65%; Sp: 66%). Conclusion: Four months after ACLR using a bone–patellar–tendon–bone procedure, the Quadriceps and Hamstring LSI associated to the Lysholm score could help make the decision to allow return to running.

## 1. Introduction

After anterior cruciate ligament (ACL) tear, surgical treatment is usually performed in athletes to return at the same level of practice [1]. Different surgical procedures can be proposed depending on the graft choice, i.e., hamstring tendon, bone–patella–tendon–bone (BPTB) or quadricipital tendon [2,3]. Knee stability results are considered similar 2 years after surgery whatever the graft choice [2]. Yet, a long rehabilitation process is necessary to recover a stable and pain-free knee able to support sport constraints without apprehension or fear of knee re-injury [4,5].

Immediately after knee surgery, an arthrogenic muscle inhibition (AMI), explained by the knee effusion, particularly affects the quadriceps muscle strength [6]. The others muscles of the operated limb and the uninvolved limb are also concerned but at a lesser degree [7,8]. This AMI is present on the surgical limb for at least one year and also depends on the occurrence of knee complication (arthrofibrosis, anterior knee pain, for instance) [9,10]. In addition, the graft site morbidity explains the quadriceps strength loss in case of BTPB procedure [11,12,13]. This strength deficit prevents patients from performing symmetrical activities such as walking, which may require knee braces to passively control knee extension during the stance phase in case of deep muscle inhibition shortly after surgery [14,15]. From the third month after surgery, running may be possible if a certain degree of symmetrical movement is possible [16,17]. Indeed, quadriceps and hamstring strengths are associated with knee kinematics and kinetics during running [18,19,20,21,22]. Significant correlations were found between isokinetic quadriceps strength and knee extension moment during running [20,23]. This ability to run represents a crucial step to continue the rehabilitation process with the final goal of returning to sport without limitation [24]. Yet, return to running without sufficient quadriceps strength recovery may lead to risk of knee complications (pain, swelling, or graft failure). So, muscle knee strength recovery monitoring could be a criterion to allow return to running or not.

Different authors have empirically proposed strength parameters to allow return to running [17]. A recovery between 65 and 80% of the quadriceps limb symmetry index (LSI) would be necessary to run safely at 3 months after ACL reconstruction (ACLR) [16]. More recently a cut-off of 60% was associated to the possibility of running at 4 months post-surgery after hamstring or BPTB graft procedure [25]. However, this cut-off depended on the graft choice [25]. At 3 months post-surgery, using hamstring graft procedure, a recent study determined the best isokinetic quadriceps strength cut-off of 1.40 Nm/kg on the surgical limb to return to running [26]. Only 53% of the patients had been able to run with a functional brace for 5 min at a speed of 9 km/h without knee pain, anxiety, or limp. To our knowledge, no specific isokinetic knee strength cut-off exists to help make the decision to allow the return to running after ACLR using the BPTB procedure. So, the objective of this study was to determine if isokinetic strength parameters could predict the return to running at 4 months after ACLR using the BPTB procedure. Indeed, we hypothesized that a significant difference in strength measures between patients who returned to running and those who did not after ACLR using BPTB procedure would provide reliable isokinetic strength cut-offs.

## 2. Materials and Methods

### 2.1. Population

**Figure 1:**
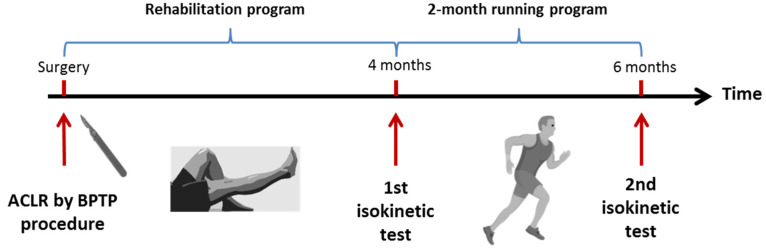
Experimental scheme of the study.

All the patients who between 2010 and 2020 had performed an isokinetic knee strength evaluation at 4 months after a primary ACLR using a BPTB procedure were included if they were followed at 6 months to validate the return to running or not (Figure 1). So, we included 216 patients (24.5 ± 5.8 year; 74 ± 11.9 kg; 175 ± 9 cm). Exclusion criteria were: patients operated upon using other ACLR graft procedures or operated upon for a posterior cruciate ligament or collateral ligaments reconstructions, patients suffering from knee effusion, knee locking, extension loss > 15°, or with limp. The patients who had already returned to running before knee evaluation or did not aim to return to running or were not followed at 4 and 6 months’ post-surgery were not included in the analysis.

The delay of ACL tear to ACLR, the sport practice, and the level of sport practice before ACL tear were recorded.

In this interventional study, necessary processes were performed with the “Direction de la Recherche Clinique” (DRC) of the University Hospital, and ethical approval was obtained from the local committee of ethics “Groupe Nantais d’Ethique dans le Domaine de la Santé” (GNEDS) on 20 May 2020. The database was anonymized. All patients gave their written consent to participate to the study without receiving any financial compensation.

### 2.2. ACLR Using a BPTB Arthoscopic Procedure

The BPTB procedure consisted in harvesting a 10-mm patellar tendon graft via a longitudinal incision [27]. The graft included a 25-mm patellar bone block and a 25-mm tibial bone block. The bone blocks were trimmed to pass through a 9-mm diameter gauge. A guide wire was drilled from the medial side of the tibial tubercule at a 55° angle to the tibial shaft and advanced to the preserved ligament trump in the posterior portion of the ACL footprint with use of a drill guide. A cannulated drill bit was used to drill the 9-mm tibial tunnel. A guide wire was drilled to perform a 25-mm depth femoral tunnel. The graft was passed into the knee and fixed with a 7 × 25 mm titanium femoral interference screw (Biosure^®^ Smith&Nephew, London, UK). The graft was tensioned to 20 pounds and fixed with another 7 × 25 mm interference screw, knee at 20° of flexion. Meniscus procedure was realized arthroscopically in accordance with the lesion or the stability of the meniscus injury. Extra-articular tenodesis with iliotibial band was added to augment ACLR in case of strongly positive pivot shift.

### 2.3. Rehabilitation Program

The rehabilitation program was a revisited accelerated program for ACLR using BPTB procedure derived from those firstly described by the team of Shelbourne [28,29]. Immediately after surgery (skin closure), contention and ice were applied on the operated leg using a game ready system^®^ (GRPro 2.1 system, Sport-Protech, Serezin-du-Rhone, France) to limit knee effusion. This treatment was performed for an average of 10 days until knee effusion disappearance. Knee brace in extension was used only if a total quadriceps muscle inhibition on the surgical limb was present. Electric quadriceps stimulation was used to treat AMI (Compex Pro Rehab^®^, Compex, France). Walking and standing were limited during the first week to decrease knee effusion. Walking was allowed with crutches until limp resolution. The recovery of knee range of motion was effective using passive and active knee extension and flexion repeated movement supervised by a physiotherapist. When the knee range of motion (ROM) was of 0 to 120 degrees, cycling was allowed 3 times a week, beginning with 15 min and continuing until 180 min or more, for 2 months.

At 4 months post-surgery, isokinetic knee strength testing was carried out to quantify the quadriceps and hamstring muscle inhibition and to precise the functional recovery using the Lysholm score. A 2-month running program was proposed to the patients who wanted to return to running (3 times a week beginning by 15 min, with an increase of 5 min per week until they could run for 30 min). The RTR protocol allowed the patient to gradually return to running, beginning with jogging (speed <10 min per mile or 10 km/h). The program was self-heart-rate-monitored to obtain 70% of the theoretical maximal heart frequency (maximal heart frequency = 220 − age). At 6 months post-surgery, the patient was assessed to know if the running program had been performed. Return to running was judged effective if the patient was able to run at least twice a week for 20 min or more.

### 2.4. Clinical Evaluation by the Lysholm Knee Scoring Scale

The Lysholm scale was created for the follow-up of ACLR [30,31]. It measures the domains of symptoms and complaints and also partially measures functioning in daily activities. This score consists of 8 items on 100 points (pts): 4 items reporting knee symptoms—pain (25 pts), giving way sensation (25 pts), knee locking sensation (15 pts), and effusion (10 pts) as well as 4 items reporting the functional capacities limp (5 pts), using crutches (5 pts), climbing stairs (10 pts), and squatting (5 pts). Higher scores indicate fewer symptoms and higher levels of functioning. The reliability, validity, and responsiveness of the Lysholm score are good or acceptable [32]. Knee complications such as arthrofibrosis, anterior knee pain, and knee effusion were reported [33,34,35].

### 2.5. Isokinetic Procedure

Knee isokinetic evaluation was performed using a Humac^®^ isokinetic dynamometer (Medimex, Sainte-Foy-lès-Lyon, France). Each subject was seated with a hip angle of 85 degrees. The mechanical axis of the dynamometer was aligned with the lateral condyle of the knee. The trunk and the thigh were stabilized with belts. The knee range of motion was 100 degrees (100 to 0 = maximal knee extension). Torque was gravity-corrected, and the dynamometer recalibration was performed monthly in accordance with the manufacturer’s instructions. Every session was preceded by familiarization with the isokinetic movements (3 submaximal movements). The patients were tested over 3 maximal repetitions at the angular speed of 60°/s followed by 5 maximal repetitions at 180°/s. A 20-s recovery period was allowed between both series. The uninvolved limb was always first evaluated after instruction and with verbal encouragement and visual feedback. All evaluation tests were conducted by the same sport physician blindly chosen from a group of surgeons. The isokinetic parameters of the study were: the peak torque of the quadriceps and the hamstring expressed in Newton-meter per kilogram (QS/BW and HS/BW in Nm/kg), the Quadriceps and Hamstring Limb Symmetry Index (Q-LSI and H-LSI) and the Hamstring-to-Quadriceps ratio (H/Q). The LSI was expressed in percentage and calculated from the formulae: peak torque of the operated limb/peak torque of the uninvolved limb) × 100 [16]. The reliability of the quadriceps and the hamstring peak torques and the SI were considered good to excellent (ICC: 0.78–0.98) [36].

### 2.6. Statistical Analysis

The statistical analysis was performed using the SPSS 23.0^®^ software package (IBM corp. Armonk, NY, USA). The quantitative variables were expressed as average and standard deviation. The categorical variables were expressed as numbers or frequencies. Two groups were established depending on the return to running (RTR) or not (NRTR). The comparison between the RTR and NRTR groups was assessed by the Student’s *t* test and the χ2 test. Multivariate analysis was used with ascendant Wald logistic binary regression (outcome: return to running or not) to identify independent predictors of return to running (inclusion of the parameters from the univariate analysis with probability < 0.10). Odds ratios [Exp(*B*)] and 95% confidence intervals (95%Cis) were estimated [37]. The Hosmer–Lemershow test was used to know if the data fitted the model well. The R-squares of Cox-Snell and Nagelkerke (% of the variance explained by the predictors) were used to know if the model was well adjusted. The cut-off value of independent predictors was chosen from the receiver operating characteristic (ROC) curve. This optimum value gave equal weight to false positive and false negative values. The ROC curve area interpretation was: excellent (>0.900), good (0.800–0.900), fair (0.700–0.800), and poor (0.600–0.700) [38]. The sensitivity, specificity, and positive and negative likelihood ratios were calculated for the cut-offs [39]. A positive likelihood ratio of 2 to 5 was considered moderately useful to predict return to running. Two statistical models were developed: one took the patient as unit (LSI) and the other the limbs (Strength/BW and H/Q ratios). Results were considered significant at the 5% critical level (*p* < 0.05). The calculation of number of variables to conduct a logistic binary regression included one explicative variable for 10 patients able to return to running [37]. For this study, a maximum of 10 explicative variables could be introduced in the model because 103 patients had returned to running.

## 3. Results

From a cohort of 315 eligible primary ACLR using BPTB graft procedure, 99 patients (31.4%) were not included for analysis because they did not provide the written consent (n = 10), they had tried to run before 4 months after surgery (n = 12), they had been evaluated after the fifth month post-ACLR (n = 17), they did not have the aim to RTR (n = 39), or they had not been followed at 6 months after ACLR (n = 21). Finally, 216 patients (24.5 ± 5.8 year; 74 ± 11.9 kg; 175 ± 9 cm) were analyzed. The delay between ACL tear and the surgery was 236 ± 367 days and the follow-up post-ACLR for isokinetic testing was 123 ± 16 days, and 103 patients (47.6%) returned to running.

### 3.1. Comparison between RTR and NRTR Groups

There was no difference between the RTR and NRTR groups for age, weight, sex, ACL tear to ACLR delay, sport, and level of practice before knee injury, tenodesis, or menici association procedures. The Lyshom score and all studied isokinetic parameters were significantly different, except the H/Q ratios in the uninvolved limb (Table 1 and Table 2). The knee complications were significantly less frequent in the RTR groups for arthrofibrosis (2.9% vs. 17.7%) and anterior knee pain (11.7% vs. 32.7%). During the 4 to 6 months follow-up, no patellar tendon graft tear was reported in the population.

From the univariate analysis, the Lysholm score and all the isokinetic parameters (LSI and strength to BW) were potentially relevant except the H/Q ratios in the uninvolved limb (Table 3). Age, sex, weight, height, ACL tear to surgery delay, surgery to isokinetic testing delay, meniscal procedures, and pre-injury Tegner Activity Scale were not relevant to be included in the multivariate analysis.

### 3.2. Predictive Model including LSI for Return to Running

**Figure 2:**
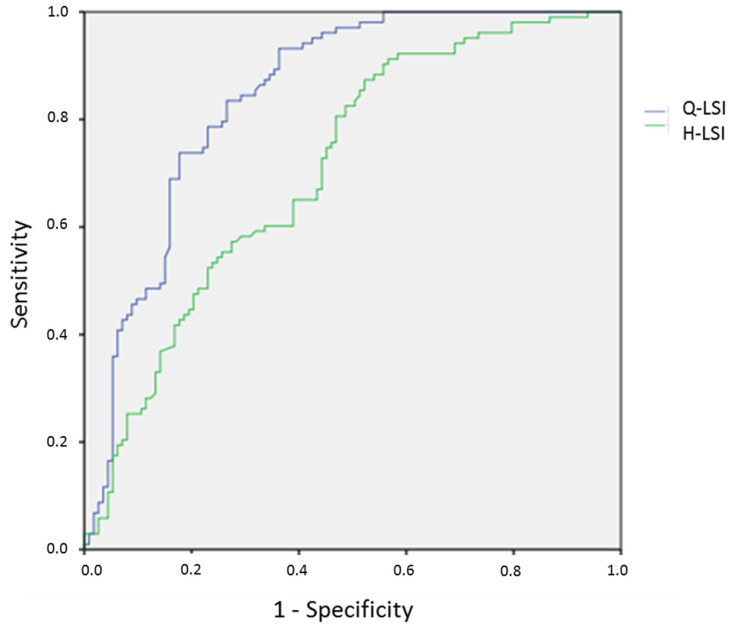
Receiver operating characteristic (ROC) curve area to return to running with Q-LSI and H-LSI. Abbreviations: Q: quadriceps; H: hamstring; LSI: Limb Symmetry Index.

The model to predict the return to running included the Lysholm score, the Q-LSI at 60°/s, and the H-LSI at 60°/s. The percentage of correct classification by hazard was of 52.6% and the prevision by the model was 77.7% (Table 4). The knee complications were not retained in the model. The ROC curve area (Figure 2), cut-off, sensitivity, specificity, and positive and negative likelihood ratio for the Lysholm score, the Q-LSI at 60°/s, and the H-LSI at 60°/s are presented in Table 5. The Q-LSI, and H-LSI cut-offs were respectively 60% and 90%.

### 3.3. Predictive Model including Strength to Body Weight for Return to Running

**Figure 3:**
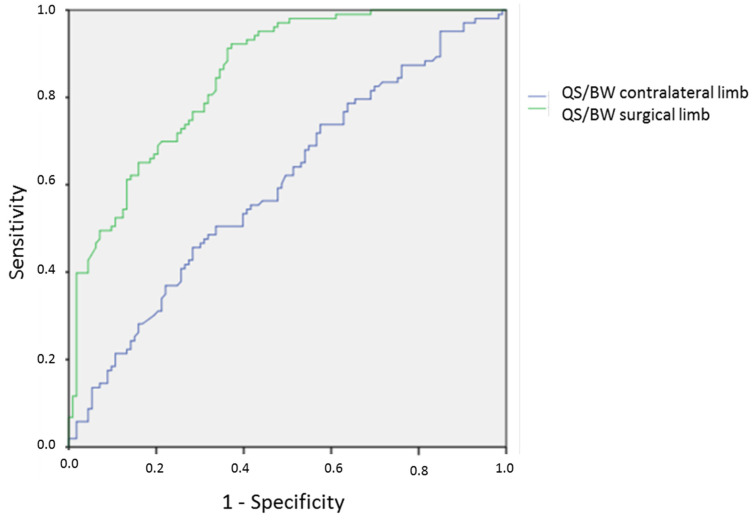
Receiver operating characteristic (ROC) curve area to return to running with QS/BW at 60°/s in surgical and contralateral knee. Abbreviations: QS: quadriceps strength; BW: body weight.

The model to predict the return to running included the QS/BW at 60°/s in surgical and uninvolved limb. The prevision by the model was 76.4% (Table 6). Knee complications, HS/BW, and H/Q ratios were not retained in the model. The ROC curve area (Figure 3), the cut-off, sensitivity, specificity, and positive and negative likelihood ratio for the QS/BW at 60°/s in surgical and uninvolved limb are presented in Table 7. The cut-off of QS/BW at 60°/s in the surgical limb was of 1.60 Nm/kg and the cut-off of QS/BW at 60°/s in the uninvolved limb was of 2.70 Nm/kg.

## 4. Discussion

The return to running represents a compulsory step to get back to “dangerous” sports for knees because of pivoting and cutting as it is the case for soccer, basketball, or handball. However, the definition of RTR is questionable. Indeed, a jogging trial with a mean speed of 9 km/h for only 5 min, and realized with a functional brace at 3 months post-ACLR using the hamstring procedure was defined RTR by Iwame et al. [26]. We have used a more ecological definition based on the frequency of more than twice a week with a 20-min running practice. This definition seems more appropriate because it does not take into account the running speed, which was found to be different from one subject to another depending on the individual aerobic capacity (70% of the target heart-rate zone) in accordance with the 2018 Physical Activity Guidelines of the American Heart Association [40]. For instance, a difference can be made for jogging according to sex: 10 min 21 s per mile for women and 9 min 3 s for men (data published in 2015 by the social network for athletes of Strava) [41]. In addition, a too-fast running speed can cause limping after ACLR due to impaired neuromuscular performances, including quadriceps strength deficit at the 4- and 6-month time points [22]. Indeed, it has been previously shown that athletes with ACLR had altered corticospinal excitability, which was related to quadriceps strength deficit, persistant from 2 weeks after ACLR to the time of return to running [42]. So, our RTR protocol allows the patient to gradually return to running, beginning with jogging (speed < 10 min per mile or 10 km/h) with a comfortable pace due to the recovery of the surgical limb.

To our knowledge, it is the first study which has explored the prediction to return to running after ACLR using the BTPB procedure. We have shown that RTR decision could be helped by the Q-LSI and the H-LSI and the Lysholm score according to the respective cut-offs of 60%, 90%, and 97 points at 4 months after ACLR using the BPTB procedure. This model seems to be more appropriate because it takes into account the knee function and the recovery of the strength of the surgical limb compared to the uninvolved limb. Firstly, the Lysholm score assesses knee complications according to symptoms (pain and effusion), and the knee range of motion is evaluated indirectly by functional parameters such as climbing stairs and squatting. The cut-off of 97 points (95%CI: 95–98 points) also corresponds to an excellent knee score (>91 points) with an absence of anterior knee pain, arthrofibrosis, or knee effusion [10,31]. Secondly, the Q-LSI calculated at 60°/s shows the difficulty for the Quadriceps strength recovery at 4 months post-surgery after BPTB procedure. Our results are comparable with those of Cristiani et al. who reported a mean Q-LSI at 60 and 65.2%, respectively, after BPTB graft procedure and standard or accelerated rehabilitation program without any significant difference between the two rehabilitation modes [13]. The originality of the Q-LSI was that this parameter could help make the decision to RTR with a cut-off of 60%. This cut-off was previously described by our team [25], but this new study confirms that this cut-off is particularly adapted for the BPTB procedure. This cut-off could authorized more patients to return to running in comparison to the 70% cut-off, the most empirically cited in the literature, without precisely knowing if this cut-off depends on the BPTB or the hamstring graft procedure [16,17]. The cut-off of 60% can be considered safe because no graft failure occurred during the return to running, and the concomitant consideration of the Lysholm score (cut-off of 97 points) avoids RTR to a patient with a significant knee complication (anterior knee pain, knee effusion, arthrofibrosis). Moreover, the quadriceps strength is the reflection of the individual quadriceps inhibition due to the evolution time of the surgical limb [35]. Thirdly, The H-LSI parameter is interesting to consider because a cut-off of 90% testifies of the recovery of hamstring strength, which is poorly affected by the BPTB graft procedure contrary to the Hamstring graft procedure [13,43]. Indeed, the absence of Hamstring graft morbidity explains the fast hamstring strength recovery after BTPB procedure. On the contrary, the arthrofibrosis complication is responsible for a poor hamstring strength LSI even in case of BPTB procedure [10,34].

The second model based on the operated upon and the uninvolved limbs is more difficult to use despite a similar prediction of RTR. The QS/BW at 60°/s in the operated upon and the uninvolved limbs could predict the return to running. This model was built because some authors questioned the interest of the strength LSI [26,44]. Indeed, the value of the LSI depends on the strength of the uninvolved limb and, the AMI also affects the uninvolved limb between 4 to 6 months post-surgery [45,46,47]. However, the quadriceps muscle strength on the operated limb is much more inhibited because of the BPTB graft morbidity [43]. Furthermore, only the operated limb displayed significant deficits of 57% for the peak torque extensor moment during a 3.6 m/s running kinetic evaluation at 4 months after the BPTB procedure [22]. At 6 months’ post-surgery, a deficit of peak rate of the quadriceps torque existed isometrically and dynamically in the operated limb at 2.7 m/s [19]. So, using the QS/BW on the surgical limb can be interesting to make the decision to allow the return to running, but using the same parameter on the uninvolved limb is more limited. Indeed, Knurr et al. have not found a significant biomechanics running change in the non-surgical knee throughout pre-injury to the 4, 6, 8, and 12 months post-surgery [22]. From an isokinetic point of view, our predictive model has shown that the Beta value was negative, which statistically showed that the weaker the QS/BW on the uninvolved limb was, higher the possibility to return to running was. According to this statistical observation, it amounts to saying that running is a symmetrical physical activity, so using Q-LSI is simpler. The cut-off value of the QS/BW on the uninvolved limb is also questionable because this performance depends on the particular characteristics of the studied population. If the frequency of sex of our population, or age, or elite sport practice was different between the two groups, for instance, and these parameters were included in the predictive model. In a general population, it has already been proved that females have less strength than males and that age or elite sport practice can influence muscle strength [48,49]. For instance, the mean quadriceps strength for handball and soccer women players aged 20 is 2.42 and 2.33 Nm/kg, respectively, and for healthy men and women aged 50, it is 2.31 and 1.91 Nm/kg, respectively [48,49]. So, the cut-off of the QS/BW of 2.70 Nm/kg in the uninvolved limb cannot be applicable for all the patients, while the LSI can always be used because this parameter is individual. Nevertheless, we have reported the QS/BW cut-off value of 1.60 Nm/kg in the surgical limb at 4 months post-ACLR. This value is higher than the value of 1.45 Nm/kg identified by Iwane et al. [26] and may be explained by the delay of 4 months. Indeed, Iwane et al. had described the QS/BW a significant indicator of safety when initiating jogging 3 months after ACLR using Hamstring graft [26].

Although our study included several parameters to establish a predictive model for RTR, some limitations can be found because some parameters have not been studied. Several studies added a lot of functional parameters to help make the decision to return to sport, such as balance tests, hop tests, and knee laxity measurements associated with isokinetic tests [50,51,52,53]. Yet, this complete evaluation is available from the sixth month after surgery, and not at 4 months [51,54]. Indeed, hop testing can be dangerous for the knee because a landing may be responsible for a graft failure [55,56,57]. So, this test is rarely proposed as early as 4 months after surgery. Balance tests could be interesting but are difficult to interpret due to measurements in different positions (bipedal or single-leg or in star position with open or closed eyes) [53]. Knee laxity can be measured with different devices but is not really interesting at 4 months after surgery because a residual knee laxity does not prevent RTR [58]. Running corresponds to a well-supported activity as shown during the functional treatment after ACL injury [18]. Another limitation is not having included a kinesiophobia score. Yet, the relationship between kinesiophobia, or fear of re-injury, evaluated with the Tampa scale and muscle activity asymmetry during gait cycle is controversial [14,59]. Nevertheless, it would be interesting to include this parameter in the future to know its influence on RTR.

## 5. Conclusions

Isokinetic muscle strength and Lysholm score can help make the decision to allow return to running at 4 months after ACLR using the BTPB procedure. The model including the Quadriceps LSI, the Hamstring LSI and the Lysholm score seemed to be the best model in clinical practice and was able to identify 77.7% of the patients in the RTR group at 4 months after surgery. Therefore, a patient with severe knee complications such as anterior knee pain or arthrofibrosis or knee effusion will be advised not to run if the different cut-offs now established are not reached (60% for Q-LSI, 90% for H-LSI, and 97 points for the Lysholm score). Furthermore, using theses cut-offs could motivate patients and decrease apprehension and fear of knee re-injury at 4 months post-ACLR. The second model, based on the quadriceps strength, seemed to be less efficient because the cut-off of the uninvolved limb (2.70 Nm/kg) was dependent on the characteristics of the patients. Some patients were not able to reach that level of muscle strength. Additionally, that model based on the strength provided arguments to guide strength symmetry during the rehabilitation process so as to return to running.

## Figures and Tables

**Table 1:** Comparison between the group of patients who returned to running (RTR group) and those who did not (NRTR group) before isokinetic measurements.

	RTR Group (n = 103)	NRTR Group (n = 113)	*p*
Age (year)	24.0 ± 6.0	24.9 ± 5.6	0.22
Sex (Male/Female)	74/29	82/31	0.90 ^a^
Body weight (Kg)	72.8 ± 9.3	75.2 ± 9.3	0.13
Body Height (cm)	175.0 ± 8.0	176.0 ± 9.0	0.31
Sport practice before ACL tear:			0.25 ^a^
Soccer	58 (56.3%)	58 (51.4%)	
Basket	14 (13.7%)	18 (15.9%)	
Handball	9 (8.7%)	8 (7.1%)	
Rugby	11 (10.7%)	5 (4.4%)	
Fighting sport	2 (1.9%)	7 (6.2%)	
Skiing	1 (0.9%)	3 (2.6%)	
Others	8 (7.8%)	14 (12.4%)	
Level of sport practice:			0.71 ^a^
Tegner score 5	8 (7.8%)	15 (13.3%)	
Tegner score 6	15 (14.6%)	17 (15.1%)	
Tegner score 7	43 (41.7%)	46 (40.7%)	
Tegner score 8	29 (28.1%)	24 (21.2%)	
Tegner score 9	6 (5.9%)	8 (7.1%)	
Tegner score 10	2 (1.9%)	3 (2.6%)	
Associated Surgery: -			0.65 ^a^
-None	79 (76.6%)	87 (77.0%)	
-Medial meniscus	14 (13.7%)	10 (8.9%)	
-Lateral meniscus	7 (6.9%)	9 (8.0%)	
-Both menici	1 (0.9%)	2 (1.7%)	
Anterolateral tenodesis	2 (1.9%)	5 (4.4%)	
ACL tear-ACLR delay (months)	250 ± 391	223 ± 344	0.58
ACLR-Isokinetic testing delay (days)	122 ± 16	122 ± 17	0.68
Lysholm score	95 ± 8	88 ± 9	<0.001

Abbreviations: ACLR: anterior cruciate ligament reconstruction; ^a^ χ2 test.

**Table 2:** Comparisons of isokinetic parameters and knee complications between the group of patients who returned to running (RTR group) and those who did not (NRTR group).

	RTR Group	NRTR Group	*p*
	(n = 103)	(n = 113)	
*Isokinetic LSI*			
-Q-LSI at 60°/s	68.5 ± 10.3	50 ± 15.5	<0.001
-Q-LSI at 180°/s	76.2 ± 8.5	60.7 ± 19.7	<0.001
-H-LSI at 60°/s	96.5 ± 12.5	85.3 ± 15.8	<0.001
-H-LSI at 180°/s	100 ± 13.1	91.6 ± 18.0	<0.001
*Isokinetic strength to Body weight*			
QS/BW at 60°/*s*:			
-Surgical limb	1.90 ± 0.38	1.31 ± 0.42	<0.001
-Uninvolved limb	2.78 ± 0.39	2.64 ± 0.39	0.008
QS/BW at 180°/s:			
-Surgical limb	1.33 ± 0.21	1.03 ± 0.29	<0.001
-Uninvolved limb	1.50 ± 0.23	1.44 ± 0.25	0.04
HS/BW at 60°/s:			
-Surgical limb	1.44 ± 0.24	1.22 ± 0.29	<0.001
-Uninvolved limb	1.33 ± 0.21	1.03 ± 0.29	<0.001
HS/BW at 180°/s:			
-Surgical limb	1.10 ± 0.19	0.95 ± 0.23	<0.001
-Uninvolved limb	1.10 ± 0.20	1.05 ± 0.22	0.05
*Isokinetic strength ratio (%)*			
H/Q at 60°/s:			
-Surgical limb	77.4 ± 14.2	101 ± 39.4	<0.001
-Uninvolved limb	54.4 ± 5.8	54.9 ± 8.5	0.58
H/Q at 180°/s:			
-Surgical limb	83.1 ± 13.2	97.6 ± 32.5	<0.001
-Uninvolved limb	63.1 ± 9.3	60.9 ± 9.6	0.1
*Knee complications*			
-None	85 (82.5%) *	51 (45.1) *	<0.001 ^a^
-Arthrofibrosis	3 (2.9%) *	20 (17.7%) *	
-Anterior knee pain	12 (11.7%) *	37 (32.7%) *	
-Knee effusion	3 (2.9%)	5 (4.4%)	

Abbreviations: Q: quadriceps; H: hamstring; LSI: Limb Symmetry Index; ^a^: χ2 test; * Significant difference between frequencies of the two groups.

**Table 3:** Predictive parameters to return to running (univariate analysis).

	*B*	Wald	OR	95% CIs	*p*
Lysholm	0.06	25	1.06	[1.03–1.08]	<0.001
Knee complications:					
-Arthrofibrosis	1.02	1.84	2.77	[0.63–12]	0.17
-Anterior knee pain	−1.38	2.09	0.25	[0.03–1.63]	0.14
-Knee joint swelling	−0.61	0.58	0.54	[0.11–2.6]	0.44
QS/BW at 60°/s:					
-Surgical limb	3.6	51.9	36	[13.7–97]	<0.001
-Uninvolved limb	0.93	6.8	2.5	[1.2–5.1]	0.009
QS/BW at 180°/s:					
-Surgical limb	4.46	41.1	87	[22–340]	<0.001
-Uninvolved limb	1.13	3.99	3.09	[1.02–9.3]	0.046
HS/BW at 60°/s:					
-Surgical limb	3	26.5	20	[6.4–62]	<0.001
-Uninvolved limb	4.46	41.1	87	[22–340]	<0.001
HS/BW at 180°/s:					
-Surgical limb	3.21	20.4	25	[6.1–100]	<0.001
-Uninvolved limb	1.25	3.61	3.5	[0.96–12]	0.056
Q-LSI at 60°/s	10.07	50.78	46390	[2415–890,872]	<0.001
Q-LSI at 180°/s	8.75	37.38	6351	[383–105,163]	<0.001
H-LSI at 60°/s	5.69	24.66	296	[31–2809]	<0.001
H-LSI at 180°/s	3.61	14.8	37	[5.9–232]	<0.001
H/Q at 60°/s:					
-Surgical limb	−4.91	31.5	0.007	[0.001–0.04]	<0.001
-Uninvolved limb	−0.97	0.3	0.37	[0.01–11.9]	0.58
H/Q at 180°/s:					
-Surgical limb	−3.46	16.3	0.031	[0.006–0.16]	<0.001
-Uninvolved limb	2.46	3.13	11.4	[0.67–204]	0.1

Abbreviations: QS: quadriceps strength; HS: hamstring strength; BW: body weight; LSI: Limb Symmetry Index; H/Q: Hamstring/Quadriceps Ratio.

**Table 4:** Predictive model to return to running from isokinetic LSI parameters.

	*B*	Wald	*p*	Exp(*B*)	95%CIs
Lysholm score	0.074	12.6	0.001	1.07	1.03–1.12
Q-LSI at 60°/s	10.13	36.4	0.001	25,098	936–672,590
H-LSI at 60°/s	3.50	5.6	0.018	33.3	1.83–606
Constant	−16.3	36.2			

Abbreviations: Q: quadriceps; H: hamstring; LSI: Limb Symmetry Index.

**Table 5:** ROC curve area, cut-off, sensitivity, specificity, and likelihood ratio for predictive parameters.

	ROC Curve Area [95%CI]	Cut-Off [95%CI]	Se	Sp	LR+	LR−
Lysholm Score	0.691 [0.621–0.762]	97 points [95–98]	65%	66%	1.91	0.53
Q-LSI at 60°/s	0.847 [0.795–0.899]	0.60 [0.58–0.62]	77.5%	77%	3.39	0.29
H-LSI at 60°/s	0.830 [0.774–0.886]	0.90 [0.87–0.93]	65.7%	61.9%	1.72	0.55

Abbreviations: Q: quadriceps; H: hamstring; LSI: Limb Symmetry Index; Se: sensitivity; Sp: specificity; LR+: positive likelihood ratio = Se/(100 − Sp); LR−: negative likelihood ratio = (100 − Se)/Sp.

**Table 6:** Predictive model to return to running from isokinetic knee parameters.

QS/BW at 60°/s	*B*	Wald	*p*	Exp(*B*)	95%CIs
Surgical limb	4.33	50.6	0.001	75.3	22–247
Uninvolved limb	−1.33	6.9	0.008	0.25	0.09–0.70
Constant	−3.38	7.7			

Abbreviations: QS: quadriceps strength; BW: body weight.

**Table 7:** Cut-off, sensitivity, specificity, and likelihood ratio for predictive knee parameters.

QS/BW at 60°/s	ROC Curve Area [95%CI]	Cut-Off (Nm/kg) [95%CI]	Se	Sp	LR+	LR−
Surgical limb	0.851 [0.802–0.900]	1.60 [1.55–1.61]	74.5%	73.5%	2.81	0.34
Uninvolved limb	0.600 [0.525–0.676]	2.70 [1.65–1.75]	56.3%	54.9%	1.24	0.80

Abbreviations: QS: quadriceps strength; BW: body weight; Se: sensitivity; Sp: specificity; LR+: positive likelihood ratio = Se/(100 − Sp); LR−: negative likelihood ratio = (100 − Se)/Sp.

## Data Availability

The data presented in this study are available on request from the corresponding author. The data are not publicly available due to ethical reasons.
